# Elucidation of the process of delayed colonic perforation after endoscopic thermal injury in a rat model

**DOI:** 10.1038/s41598-026-45443-y

**Published:** 2026-03-22

**Authors:** Takahiro Sakae, Hidehito Maeda, Fumisato Sasaki, Yuko Nakamura, Naohiro Koyoshi, Shohei Uehara, Akihito Tanaka, Makoto Hinokuchi, Shiho Arima, Shinichi Hashimoto, Shuji Kanmura, Akio Ido

**Affiliations:** Department of Digestive and Lifestyle Diseases, Kagoshima University Graduate School of Medical and Dental Sciences, 8-35-1 Sakuragaoka, Kagoshima, 890-8544 Japan

**Keywords:** Delayed colonic perforation, Endoscopic thermal injury, Bacterial infections, Diseases, Gastroenterology, Medical research, Microbiology

## Abstract

Delayed perforation is a rare but serious complication following endoscopic resection of colonic lesions; however, its pathogenesis remains unclear. We hypothesized that its mechanism mirrors Jackson’s burn model, where a zone of stasis progresses to a zone of coagulation, ultimately leading to perforation. In this study, thermal injury was endoscopically induced in rat colons, and histopathological changes were analyzed at 12-hour intervals. To assess the role of bacterial infection, pseudo-germ-free rats were established by administering a course of antibiotics. The incidence of delayed colonic perforation was compared between the control and pseudo-germ-free rats. Degeneration and thinning of the muscularis propria persisted for 36 hours post-injury, with destruction of this layer observed at 48 hours. Dilated vessels in the mucosa and submucosa peaked at 24 hours, forming a zone of stasis. By 36 hours, mucosal necrosis had intensified, myeloperoxidase-positive cells had increased in the submucosa, and *Escherichia coli* had translocated into deeper layers, coinciding with expansion of the zone of coagulation. Moreover, pseudo-germ-free rats exhibited significantly fewer perforations. These findings suggest that delayed colonic perforation follows a pathological process similar to thermal burns and that bacterial infection accelerates this progression. Therefore, local infection control may be essential for preventing this serious complication.

**Supplementary Information:**

The online version contains supplementary material available at 10.1038/s41598-026-45443-y.

## Introduction

Endoscopic mucosal resection and endoscopic submucosal dissection (ESD) are well-established and widely used treatment modalities for colorectal tumors^1^. Although these techniques have demonstrated excellent efficacy, complications, such as intra- and postoperative bleeding or perforation, remain significant concerns^1–4^. Delayed perforation is one of the serious postoperative complications.

According to the Japan Gastroenterological Endoscopy Society’s guidelines for colorectal endoscopic submucosal dissection/endoscopic mucosal resection, delayed perforation is defined as colonic perforation detected following withdrawal of the endoscope, rather than during the procedure itself^1^. The reported incidence of delayed perforation after ESD based on large-scale retrospective and prospective cohort studies ranges from 0.1% to 2.2%^1,3,5–7^. Although rare, delayed perforation can lead to fatal clinical outcomes and often requires surgical intervention^1,3,5,7–9^.

Delayed perforation is hypothesized to result from excessive thermal injury to the submucosa and muscularis propria^4,10^; however, the exact sequence of pathological changes remains unclear. However, for skin burns, the Jackson burn model is well-established and explains the histological progression of thermal injury. Specifically, following thermal damage, a “zone of stasis” forms around a central “zone of coagulation.” The zone of coagulation consists of necrotic tissue, whereas the zone of stasis is potentially salvageable. With appropriate treatment, tissue in the zone of stasis can recover and prevent necrosis; however, without it, this zone may progress into a zone of coagulation, resulting in a deeper burn injury^11^. We hypothesized that the mechanisms underlying the progression of skin burn to deeper tissue injury are similar to those that lead to deepening and ultimately perforating colonic thermal injury. Therefore, this study aimed to elucidate the mechanisms underlying delayed colonic perforation.

## Results

### Investigation of the delayed colonic perforation process

#### Macroscopic changes over time following thermal injury

**Fig 1:**
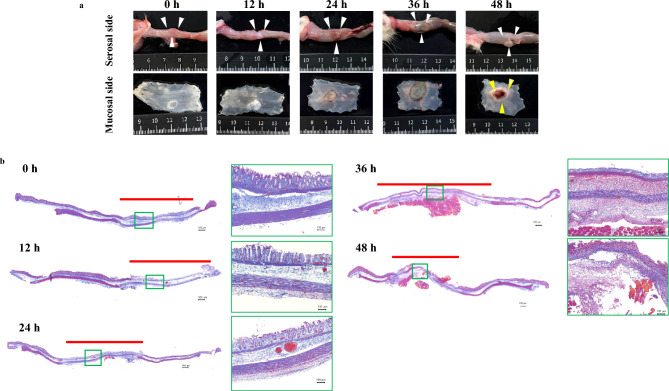
Macroscopic changes over time following thermal injury. (**a**) The white arrowhead indicates the area of thermal injury. The serosal surface showed discoloration suggestive of decreased blood flow, and by 48 hours, an abscess had formed. On the mucosal surface, the ulcer progressively deepened, leading to perforation by 48 hours (yellow arrowhead). (**b**) The muscularis propria showed degeneration and thinning immediately after thermal injury, which persisted for 36 hours before disappearing by 48 hours.

The mucosal epithelium appeared white immediately after thermal injury. At 12 hours post-injury, a whitish discoloration was observed on the serosal surface. At 24 hours, a brownish discoloration, suggestive of decreased blood flow, was observed on the serosal surface. On the mucosal surface, the epithelium had sloughed off, and an ulcer had formed. At 36 hours, the brownish discoloration on the serosal surface was more pronounced, and the mucosal ulcer had deepened. At 48 hours, an abscess had formed on the serosal surface, and perforation was evident (Fig. 1a).

### Changes in the muscularis propria and delayed perforation rate

To track changes in the muscularis propria from initial thermal injury to delayed perforation, tissue sections were evaluated using Azan staining. The red line indicates the area of thermal injury. Degeneration and thinning of the muscularis propria were observed immediately after thermal injury and persisted for 36 hours. By 48 hours, however, the muscularis propria had completely disappeared (Fig. 1b). The delayed perforation rate was 33.3% at 36 hours and reached 100% at 48 hours (Table 1).

**Table 1:** Rate of delayed colonic perforation over time.

Time post-injury (h)	Delayed perforation rate (%)
0	0
12	0
24	0
36	33.3
48	100

### Area of dilated vessels in the mucosa and submucosa

**Fig 2:**
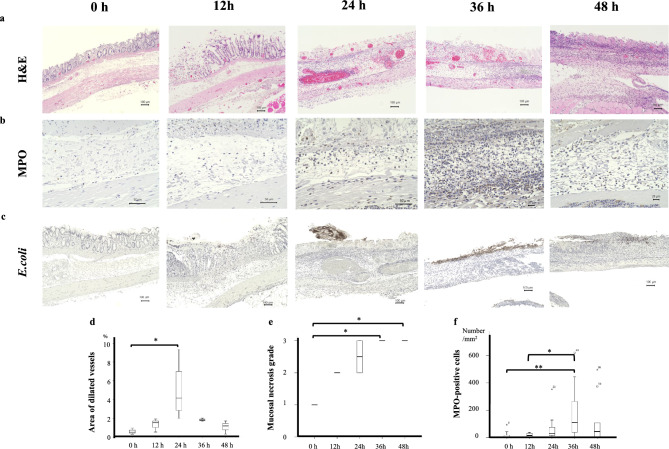
Histopathological changes over time following thermal injury. (**a**) Hematoxylin–eosin staining showing overall histopathological changes. (**b**) Myeloperoxidase (MPO) staining showing neutrophil infiltration in the submucosa. (**c**) *E. coli* staining showing bacterial infiltration; infiltration of *E. coli* into the submucosa was observed at 36 hours post-injury, coinciding with the rapid increase in MPO-positive cells. (**d**) At 24 hours post-injury, marked blood vessel congestion was observed, analogous to the zone of stasis in skin burns. (**e**) Although the glandular structure of the mucosa remained intact immediately after thermal injury, it progressively deteriorated and had completely disappeared by 36 hours post-injury. (**f**) MPO-positive cells in the submucosa increased rapidly at 36 hours post-injury, coinciding with mucosal necrosis.

To determine whether colonic mucosal findings were analogous to the zone of stasis in skin burns, we measured the cross-sectional area of dilated vessels in the mucosa and submucosa was measured over time. The cross-sectional area of dilated vessels in the mucosa and submucosa increased over time, peaking significantly at 24 hours before subsequently decreasing (0 hour vs. 24 hours; 0.594% vs. 4.920%, p = 0.043) (Fig. 2a, d).

### Assessment of thermal injury progression

As the mucosal layer likely contributes significantly to the transition from the zone of stasis to the zone of coagulation, we evaluated the extent of mucosal necrosis over time. The extent of mucosal necrosis was graded as follows: 1, mild; 2, moderate; and 3, severe. Immediately after the thermal injury, the glandular structures of the mucosa remained, but they deteriorated over time; by 36 hours, the glandular structures had disappeared (grade 1 at 0 hour vs. grade 3 at 36 hours, p = 0.032) (Fig. 2a,e).

The MPO-positive cell count in the submucosa was evaluated over time to track the course of neutrophil infiltration associated with mucosal necrosis. The number of MPO-positive cells in the submucosa increased rapidly at 36 hours, following the mucosal necrosis described above (0/mm^2^ at 0 hour and 15.10/mm^2^ at 12 hours vs. 110.5/mm^2^ at 36 hours; p = 0.006 and p = 0.032, respectively) (Fig. 2 b,f).

### Changes in E. coli staining

*E. coli* staining was used to evaluate bacterial infiltration resulting from mucosal necrosis. The submucosal translocation of *E. coli* was observed 36 hours after the onset of mucosal necrosis (Fig. 2 c).

### Assessment of the effect of colonic pseudo-sterilization on delayed colonic perforation following thermal injury

#### Gut microflora assessment in pseudo-germ-free rats

**Fig 3:**
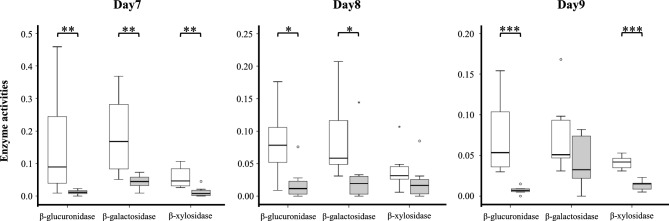
Gut microflora assessment in pseudo-germ-free rats. White columns represent the control group; gray columns represent the pseudo-germ-free group. After five consecutive days of antibiotic administration by gavage, the treatment was paused for one day. The enzymatic activities of β-glucuronidase, β-galactosidase, and β-xylosidase were then measured on days 7, 8, and 9. On day 7, the enzyme activities of all three enzymes were significantly reduced in the pseudo-germ-free group compared to the control group. On day 8, the activities of β-glucuronidase and β-galactosidase remained significantly lower in the pseudo-germ-free group. On day 9, the activities of β-glucuronidase and β-xylosidase were still significantly reduced in the pseudo-germ-free group.

After five consecutive days of antibiotic administration by gavage, the treatment was paused for one day. The enzymatic activities of β-glucuronidase, β-galactosidase, and β-xylosidase were then measured on days 7, 8, and 9. On day 7, the activity levels of all three enzymes were significantly lower in the pseudo-germ-free group than in the control group (β-glucuronidase: 0.090 vs. 0.012, p = 0.003; β-galactosidase: 0.168 vs. 0.045, p = 0.001; and β-xylosidase: 0.047 vs. 0.008, p = 0.002, respectively). On day 8, the activity levels of β-glucuronidase and β-galactosidase remained significantly lower in the pseudo-germ-free group than in the control group (0.079 vs. 0.012, p = 0.010 and 0.059 vs. 0.020, p = 0.010, respectively). On day 9, the activity levels of β-glucuronidase and β-xylosidase were still significantly lower in the pseudo-germ-free group than in the control group (0.054 vs. 0.007, p < 0.001 and 0.042 vs. 0.015, p < 0.001, respectively) (Fig. 3).

### Effect of colonic pseudo-sterilization on delayed colonic perforation after thermal injury

**Fig 4:**
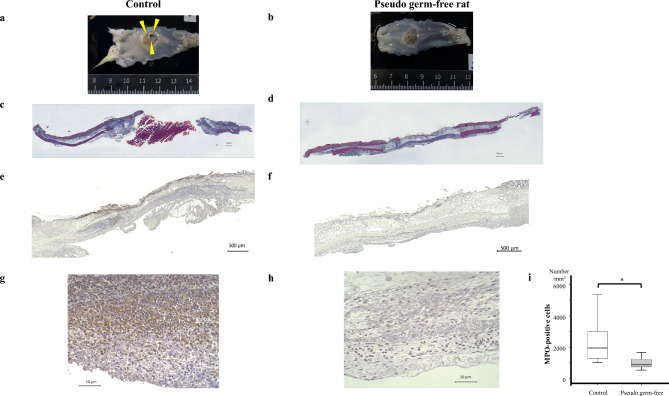
Effect of colonic pseudo-sterilization on delayed perforation following thermal injury. (**a,b**) Ulcers formed in both groups by 48 hours post-injury. However, while the control group exhibited severe inflammation and perforation (yellow arrowhead), no perforation was observed in the pseudo-germ-free group. (**c,d**) Azan staining revealed degeneration of the muscularis propria in pseudo-germ-free rats, though the layer was not completely lost. In contrast, in control rats, the muscularis propria was degenerated, necrotic, and absent. (**e,f**) *E. coli* staining revealed bacterial infiltration into the submucosa only in the control group. (**g,h,i**) The number of myeloperoxidase-positive cells in the serosa was significantly lower in the pseudo-germ-free group than in the control group.

The rats were euthanized, and their colons were excised and evaluated for perforation. The rate of delayed perforation was significantly lower in the pseudo-germ-free group than in the control group (100% vs. 11.1%, p < 0.001) (Table 2; Fig.4a, b). Azan staining was used to evaluate changes in the muscularis propria. In pseudo-germ-free rats, degeneration of the muscularis propria was observed, but the layer had not disappeared. In contrast, the muscularis propria in the control group was degenerated, necrotic, and absent (Fig. 4c, d). Furthermore, *E. coli* staining revealed infiltration into the submucosa only in the control group (Fig. 4e, f). The number of MPO-positive cells infiltrating the serosa was quantified. The number of MPO-positive cells in the serosa was significantly lower in the pseudo-germ-free group than in the control group (2304/mm^2^ vs. 987.0/mm^2^, p = 0.023) (Fig. 4g, h, i).

**Table 2:** Rate of delayed colonic perforation in control versus pseudo-germ-free groups.

	Control group	Pseudo-germ-free group	p
Delayed perforation rate (%)	100	11.1	0.000

## Discussion

Although delayed perforation is a rare complication of endoscopic resection (ER) for colonic lesions, its potentially fatal outcomes make it a significant clinical concern^1,3,5,7–9^. Delayed perforation is hypothesized to result from excessive thermal injury to the submucosa and muscularis propria^4,10^. Furthermore, infection has also been reported as a potential cause of delayed perforation^9^. However, the precise mechanisms remain unclear. Therefore, elucidating the mechanism underlying delayed colonic perforation after ER is essential for developing effective preventive strategies.

Although the precise mechanism underlying delayed colonic perforation is not fully understood, the progressive pathology of thermal skin injury has been well-characterized by the Jackson burn model^11^. In burn treatment, it is crucial to prevent the “zone of stasis”—an area of critically damaged but potentially salvageable tissue—from progressing to the “zone of coagulation,” where irreversible necrosis occurs^11^. Infusion therapy, infection control, and local wound care play essential roles in this process^11^. If delayed colonic perforation follows a pathological progression similar to that of the Jackson burn model, dermatological insights could help prevent the progression of thermal injury in the colon.

**Fig 5:**
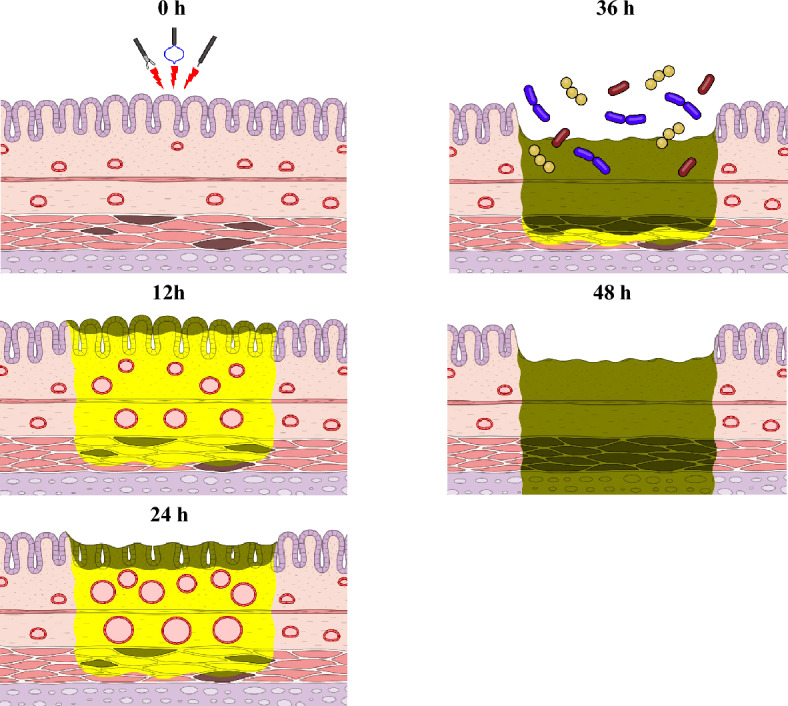
Process of delayed colonic perforation following endoscopic thermal injury. Similar to Jackson’s burn model, excessive thermal injury to the colon leads to the formation of zones of coagulation (brown area) and stasis (yellow area). Subsequent epithelial damage and bacterial translocation drive the expansion of the zone of coagulation. Preventing this bacterial invasion may therefore be critical to avoiding delayed perforation.

To investigate this hypothesis, we examined the temporal progression of delayed colonic perforation and found that it follows a similar course to that described in the Jackson burn model. Notably, 24 hours after thermal injury, blood flow stasis was observed, confirming the formation of a “zone of stasis.” Complete necrosis of the mucosal epithelium was observed 36 hours post-injury, at which point this zone began to transition into a “zone of coagulation.” Interestingly, during this period, we observed *E. coli* infiltration into the submucosa (Fig. 5). The mucosal epithelium was previously reported to play a crucial role in preventing bacterial invasion^12^. This raises the question of why colorectal post-ESD ulcers do not frequently lead to severe infections despite the complete removal of the mucosa. Based on our previous research in porcine models, we have confirmed that careful management of electrosurgical settings during ESD prevents excessive heat transfer to deeper layers, thereby preserving a sufficient number of blood vessels in the ulcer base^13,14^. This vascular preservation may contribute to maintaining local immune function. However, as demonstrated in this study, if thermal injury extends to the muscularis propria, most of the vasculature may be disrupted, impairing the migration of immune cells. Furthermore, systemic antibiotic prophylaxis may not be an effective preventive strategy for delayed perforation because the drug delivery to the thermally injured tissue may be insufficient. Similar to delayed colonic perforation, post-polypectomy electrocoagulation syndrome and post-endoscopic submucosal dissection coagulation syndrome (PECS) are complications resulting from thermal injury to the intestinal wall caused by electrocoagulation during endoscopic treatment^1,2,15^. Some cases of PECS may progress to delayed perforation^4,16,17^. Intravenous antibiotic administration has been explored as a preventive strategy for PECS; however, the literature is conflicting, with some reports indicating a reduction in incidence and others showing no significant effect^16,18^. Based on our findings, intravenous antibiotic administration may not be an effective strategy. Nevertheless, further studies are warranted to establish effective preventive measures for PECS.

To further assess the role of bacterial infection in delayed colonic perforation, we evaluated the impact of colonic pseudo-sterilization. Remarkably, the incidence of delayed colonic perforation in the pseudo-germ-free group was only 11.1%, which was significantly lower than the 100% incidence in the control group. These results suggest that infection prevention at the injury site is crucial for preventing delayed colonic perforation.

Although this study focused primarily on infection, we did observe one case of delayed colonic perforation in the pseudo-germ-free group. Fecal analysis confirmed that this rat exhibited substantially reduced enzyme activity, consistent with a pseudo-germ-free state. This rat exhibited hematochezia, and blood clots were observed at the ulcer base, suggesting that ischemia may have also been a contributing factor. Although infection was confirmed as a primary driver of delayed colonic perforation, further investigation is needed to clarify the role of other contributing factors.

Moreover, antibiotic treatment may have altered stool consistency and intestinal motility, potentially reducing intraluminal pressure and thereby influencing the incidence of perforation. Although intraluminal pressure was not measured in this study, its potential contribution cannot be excluded. From a clinical perspective, timely colonic drainage has been proposed as an effective strategy for managing endoscopy-related perforation^19^. Accordingly, the interaction between infectious and mechanical factors warrants further investigation.

Overall, considering the central role of bacterial translocation demonstrated in this study, local infection control at the ulcer base may serve as a more rational preventive strategy than systemic antibiotic prophylaxis alone. In light of the absence of any established effective preventive method for delayed colonic perforation, our findings provide a mechanistic rationale for the development of novel topical antibacterial approaches.

This study has several limitations. First, the rat colonic wall is substantially thinner than the human colonic wall, and our experimental model did not replicate key procedural elements of colorectal ESD, such as submucosal injection or the use of intermittent soft coagulation settings. These anatomical and procedural differences may limit the direct applicability of our findings to routine clinical practice. Therefore, the applied thermal injury should be interpreted as a simplified and standardized insult rather than a direct simulation of conventional human ESD. Moreover, this model was intentionally designed to represent an enhanced or “worst-case” injury scenario to reliably reproduce the biological cascade that triggers delayed perforation. Our aim was not to replicate the ESD procedure per se but rather to establish a reproducible mechanistic framework for investigating pathological progression. Notably, the sequential histopathological changes observed in this model mirrored the classical Jackson burn progression, supporting the biological relevance of the injury cascade. Future translational studies incorporating clinically relevant electrosurgical conditions and submucosal injection techniques will be necessary to further validate these findings. Second, a previous rat model study that systematically evaluated soft coagulation at 40 W for 2–4 seconds demonstrated that a 4-second application reliably induced delayed microperforation, whereas 2–3 seconds resulted in transmural necrosis without overt perforation^20^. Therefore, we applied soft coagulation for 4 seconds to ensure consistent progression to delayed perforation for mechanistic analysis. Future studies comparing graded injury durations may further clarify the threshold between transmural necrosis and perforation and enhance the translational relevance of this model. Third, although care was taken to ensure technical consistency when creating thermal injuries with hemostatic forceps, slight variations may have occurred due to factors such as differences in the forceps’ contact area and the tissue’s moisture content. Nevertheless, as the injuries were induced in the pseudo-germ-free and control groups under blinded conditions, these variations are unlikely to have significantly influenced the results. Fourth, in the study aimed at elucidating the pathophysiological mechanism of delayed colonic perforation through time-dependent histopathological analysis, two rats died between 24 and 36 hours after thermal injury, which precluded histological evaluation in these cases. These latter two deaths may have impacted the data. Furthermore, during our investigation into the effects of pseudo-sterilization of the colon on delayed colonic perforation after thermal injury, two rats died immediately after anesthesia, which could possibly be attributed to the deterioration of their physical condition due to 5 days of forced administration of antibiotics and sterile water. Fifth, during out investigation into the delayed colonic perforation process, the primary objective was to observe time-dependent changes. To minimize procedural variability, all thermal injuries were induced on the same day. This approach consequently limited the number of animals that could be used, resulting in a small sample size for each time point. However, the study objective (i.e., to evaluate histological changes analogous to those described in the Jackson burn model) was sufficiently achieved. Sixth, given that a germ-free large rat model was not feasible for this study due to its limited availability, a pseudo-germ-free rat model was employed. Although broad-spectrum antibiotics significantly suppressed most bacterial populations, complete elimination of bacteria was not achieved. Hence, the findings of this study reflect the impact of a substantial reduction in bacterial load, rather than absolute sterility. Further investigations using a completely germ-free model are warranted to validate these results. Sixth, this study evaluated a pseudo-germ-free state based on enzymatic activity, with our findings confirming a sustained reduction in bacterial activity up to day 9. Although not performed in this study, direct quantification of bacterial load using 16S rRNA quantitative PCR could have further validated the pseudo-germ-free condition.

## Materials and methods

**Fig 6:**
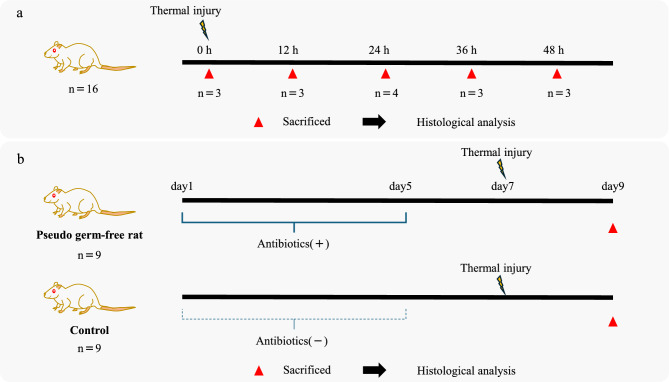
Study design. (**a**) Investigation of the pathological process underlying delayed colonic perforation. (**b**) Evaluation of the effects of colonic pseudo-sterilization on delayed colonic perforation after thermal injury.

To elucidate the pathophysiological mechanism underlying delayed colonic perforation, we used a rat model to investigate the time-dependent histopathological changes following colonic thermal injury. We focused on the transition from the zone of stasis to the zone of coagulation, as described in the Jackson burn model, to determine whether this progression contributed to the onset of delayed perforation. Among the 20 rats used in this study, 2 died during the acclimation period prior to the experiment. Moreover, two other rats were found dead between 24 and 36 hours after thermal injury. Necropsy revealed no gross evidence of perforation; however, due to postmortem rigor mortis, rectal sampling was not feasible, and discoloration of the intestinal tract was observed. Therefore, these two cases were excluded from histological evaluation (Fig. 6a). Thereafter, we evaluated fecal enzymatic activity in eight control rats and eight pseudo-germ-free rats to assess gut microflora in the pseudo-germ-free state. Finally, we sought to demonstrate that necrosis of the mucosal layer permits bacterial invasion, which facilitates the transition from the zone of stasis to the zone of coagulation within the submucosa and muscularis propria, ultimately leading to delayed colonic perforation. Therefore, we used pseudo-germ-free rats to assess bacterial contribution to the development of delayed colonic perforation. Of the 20 rats used in this study, 2 died shortly after anesthesia induction. Therefore, the analysis was conducted using nine rats in the control group and nine rats in the pseudo-germ-free group (Fig. 6b).

### Experimental animals

Fifty-seven male Sprague–Dawley rats (400–450 g) were purchased from Jackson Laboratory Japan, Inc. (Kanagawa, Japan) and housed in autoclaved, ventilated cages in a controlled environment (25°C, 50% ± 5% humidity, 12-hour light/dark cycle) with autoclaved bedding and feed. Feed and water were provided ad libitum. The rats were maintained under specific-pathogen-free conditions with free access to a standard diet and drinking water. All animal procedures were approved by the Animal Ethics Committee of Kagoshima University (approval no.: MD23022; date: June 19, 2023; approval no.: MD23100; date: April 18, 2024).

For all procedures, general anesthesia was induced by intraperitoneal injection of ketamine hydrochloride (75 mg/kg) combined with medetomidine hydrochloride (1 mg/kg). Buprenorphine (0.01 mg/kg, intraperitoneally) was administered for analgesia. Rats were euthanized under deep anesthesia by intraperitoneal overdose of sodium pentobarbital (100–200 mg/kg) and were subsequently placed in a euthanasia chamber, in which carbon dioxide was introduced at a displacement rate of 30%–70% of the chamber volume per minute until death was confirmed.

### Endoscopy

A BF TYPE 1T260 bronchoscope (Olympus, Tokyo, Japan) with an outer diameter of 5.9 mm and a 2.8-mm working channel, connected to an EVIS LUCERA ELITE video system (Olympus, Tokyo, Japan), was used for colonoscopy in this study.

### Model of delayed colonic perforation after thermal injury

Following a previously reported method, we created a rat model of delayed perforation occurring 48 hours post-injury^20^. After a 12-hour fast with free access to drinking water, the rats were anesthetized with an intraperitoneal injection of ketamine hydrochloride (75 mg/kg) and medetomidine hydrochloride (1 mg/kg) and placed in a supine position. The remaining feces were flushed out by injecting saline through the anus. The endoscope was then gently inserted through the anus. The colon was occasionally inflated with air to improve lumen visualization. A thermal injury was created in the colon, 6 cm from the anal margin using RAICHO Hemostatic Forceps (Kaneka Medix, Osaka, Japan) with a soft coagulation setting of 40 W for 4 seconds (ESG-100, Olympus, Tokyo, Japan). This technique induces deep thermal damage within 48 hours (acute phase) (Supplemental Fig 1). Delayed perforation was defined as the presence of a transmural defect at the injury site identified macroscopically on necropsy or as histopathological disappearance of the muscularis propria on Azan-stained sections.

### Assessment of gut microflora in the pseudo-germ-free state

The pseudo-germ-free state was induced in rats using a multidrug antibiotic regimen. An antibiotic cocktail in sterile water containing metronidazole (200 mg/kg), ampicillin (200 mg/kg), vancomycin (100 mg/kg), and neomycin (200 mg/kg) (all from FUJIFILM Wako, Osaka, Japan) was administered orally by gavage to the pseudo-germ-free group twice daily for 5 days^21,22^. The antibiotic mixture was vortexed thoroughly before each gavage. During this period, the rats received sterile food and water. Each rat was housed in a separate cage, and the cages were changed daily to ensure cleanliness.

The status of the gut microflora was confirmed by measuring fecal enzymatic activity in pseudo-germ-free rats using a previously described method^23,24^. Fecal samples were collected on days 7, 8, and 9 after the initiation of antibiotic treatment. The wet weight of the collected feces was determined, and the enzymatic activity was measured immediately. Then, approximately 100 mg of the fecal sample was suspended in a 20-fold volume of ice-cold saline. The fecal suspension was centrifuged at 5,000 ×g for 5 min, and the resulting supernatant was used for the enzymatic activity assay. The activities of β-glucuronidase, β-galactosidase, and β-xylosidase were measured as follows: a 0.5 mL reaction mixture contained 0.2 mL of substrate, 0.1 mL of fecal sample, and 0.2 mL of 0.1 M phosphate buffer, pH 7.0 (FUJIFILM Wako, Osaka, Japan). The substrates used were 1 mM p-nitrophenyl-β-D-glucuronide for β-glucuronidase, 1 mM o-nitrophenyl-β-D-galactoside for β-galactosidase (both from FUJIFILM Wako, Osaka, Japan), and 1 mM p-nitrophenyl-β-D-xylopyranoside for β-xylosidase (BLD Pharmatech, Shanghai, China). The reaction mixture was incubated at 37 °C for 15 min. The reaction was then stopped by adding 0.5 mL of 0.5 N NaOH (FUJIFILM Wako, Osaka, Japan), and the mixture was centrifuged at 5,000 ×g for 10 min. Absorbance was measured at 405 nm using an iMark Microplate Reader (Bio-Rad Laboratories, Hercules, CA, USA).

### Investigation of the delayed colonic perforation process

Thermal injuries in the colons of rats in the delayed colonic perforation model group were created on the same day, and the animals were euthanized at predefined time points (0, 12, 24, 36, and 48 hours after injury) for histological evaluation. We first examined macroscopic changes over time following thermal injury to assess changes in the muscularis propria and the delayed perforation rate. Next, we investigated whether findings analogous to the zone of stasis in cutaneous burns could be observed in the colonic mucosa. To achieve this, we analyzed time-dependent changes in the number of congested vessels within the mucosa and submucosa.

To further assess the progression of thermal injury, the severity of mucosal necrosis was categorized into three grades based on the percentage of mucosal epithelial loss: mild (<25%), moderate (25%–75%), and severe (>75%). Subsequently, time-dependent changes in the number of myeloperoxidase (MPO)-positive cells in the submucosa were examined. Finally, to assess bacterial infection at the injury site, time-dependent changes in *E. coli* staining were examined (Fig. 6a).

### Assessment of the effect of colonic pseudo-sterilization on delayed colonic perforation following thermal injury

Pseudo-germ-free rats and control rats (administered sterile water) underwent colonic thermal injury on day 7 induced via endoscopy with hemostatic forceps. On day 9, the colons were excited for histological evaluation. The assessed parameters included the delayed perforation rate, MPO-positive cell count in the serosa, and changes in *E. coli* staining (Fig. 6b).

### Histological analysis

After the specimens were fixed for 48 hours in 4% paraformaldehyde phosphate buffer solution (FUJIFILM Wako, Osaka, Japan), each lesion was sectioned at 4 mm intervals, embedded in paraffin, sectioned at 2 μm thickness, and stained with hematoxylin–eosin (H&E), Azan stain, or processed for immunohistochemical staining. Azan staining was used to evaluate muscularis propria damage.

### Immunohistochemistry

Immunohistochemical staining was performed using a standard enzyme-labeled antibody method. Neutrophils were identified using a rabbit polyclonal anti-myeloperoxidase antibody (1:200 dilution; Abcam, Cambridge, MA, USA). To evaluate inflammation in the submucosa and serosa, four random fields of view per section were captured (400× magnification), and MPO-positive cells were counted using ImageJ software (version 1.54f; National Institutes of Health, Bethesda, MD, USA). *E. coli* staining was used to assess bacterial invasion into the submucosa. Staining was performed using an anti-*E. coli* LPS antibody (1:500 dilution; Abcam, Cambridge, MA, USA).

### Statistical analysis

The area of dilated vessels in the mucosa and submucosa was normally distributed and therefore analyzed using Tukey’s multiple comparison test. In contrast, the extent of mucosal necrosis and the number of MPO-positive cells in the submucosa did not follow a normal distribution and were evaluated using the Steel–Dwass test. Enzymatic activity data did not conform to a normal distribution and were compared using the Mann–Whitney U test. Conversely, the number of MPO-positive cells in the serosa of the pseudo-germ-free and control groups followed a normal distribution and was compared using Student’s t-test. The incidence of delayed colonic perforation between the control and pseudo-germ-free groups was compared using Fisher’s exact test. Statistical significance was set at p < 0.05 (*p < 0.05, **p < 0.01, ***p < 0.001). Statistical analyses were performed using IBM SPSS Statistics software (version 22; IBM Corp., Armonk, NY, USA).

To assess the effects of colonic pseudo-sterilization on delayed colonic perforation following thermal injury, 10 animals were initially allocated to each group based on feasibility and ethical considerations in accordance with the 3Rs principle. Two animals died during anesthesia induction before thermal injury and were excluded from further analysis, resulting in a final sample size of nine animals per group.

With a two-sided α of 0.05, a sample size of nine animals per group provides >90% power to detect a reduction in perforation rate from 100% to 50%. Based on the observed rates in this study (100% vs. 11.1%), the estimated power exceeds 99%.

Time-course histopathological analyses were descriptive in nature and were not independently powered. Analyses of fecal enzymatic activity were exploratory and were not used to define the primary endpoint.

## Conclusions

We demonstrated that colonic thermal injury follows a course analogous to the Jackson burn model, ultimately leading to delayed perforation. Furthermore, we identified bacterial infection as a major contributing factor to this process. Based on these findings, we are currently investigating whether topical antibiotic-containing dressings, similar to those used in burn treatment, may be effective in preventing this complication. Elucidating the mechanisms underlying delayed perforation may facilitate the development of effective preventive measures.

## Supplementary Information

Supplementary Information

## Data Availability

Data is provided within the manuscript.

## Abbreviations

ER: Endoscopic resection
ESD: Endoscopic submucosal dissection
MPO: Myeloperoxidase
E.coli: Escherichia coli
H&E: Hematoxylin–eosin
PECS: Post-endoscopic submucosal dissection coagulation syndrome
