# Supplementary figures and images for: Elucidation of the process of delayed colonic perforation after endoscopic thermal injury in a rat model
Source: Sci Rep. 2026 Mar 22;16:14538. doi: 10.1038/s41598-026-45443-y (PMC13153210; doi:10.1038/s41598-026-45443-y)

## Slide 1
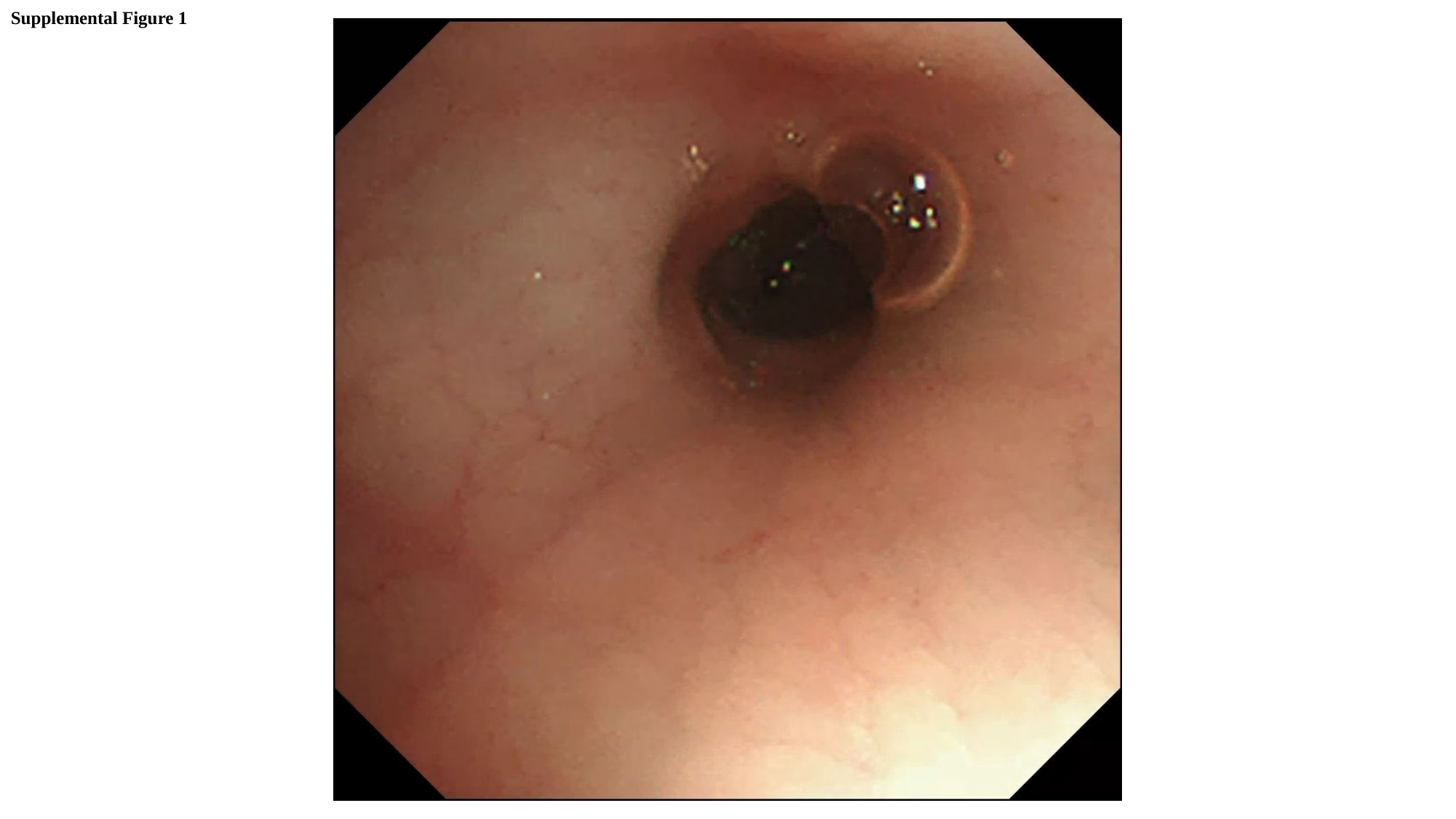

Supplemental Figure 1

Supplement: Supplementary file 1 — Supplementary Information [file 41598_2026_45443_MOESM1_ESM.pptx]
